# Disability During the Last Years of Life Among Nonagenarians: The Vitality 90+ Study 2001-2014

**DOI:** 10.1093/geroni/igaa057.745

**Published:** 2020-12-16

**Authors:** Linda Enroth, Pheck Hwa Soo, Lily Nosraty, Kristina Tiainen, Jani Raitanen, Marja Jylhä, Mari Aaltonen

**Affiliations:** 1 Tampere University, Tampere, Finland; 2 JGZ Kennemerland, Velserbroek, Netherlands

## Abstract

Increasing life expectancy has postponed the last years of life to older ages. Previous studies have demonstrated that disability is determined by age, age at death and closeness to death but only few have focused on oldest old population. We examined disability during the last years of life among people aged 90 years and older between 2001 and 2014 and assessed whether it varied by age at death, sex and study year. We used population-based survey data from the Vitality 90+ Study years 2001, 2003, 2007, 2010 and 2014 (N=5711, response rate 77-86%) linked with dates of death from Statistics Finland. Disability was defined as dependency in daily activities (dressing, getting in and out of bed) and mobility (moving indoors, walking 400m, using stairs). We analyzed disability stratified by closeness to death and age at death for men and women in each study year with logistic regression method. Disability in daily activities and mobility increased systematically with closeness to death (>4, 3-3.99, 2-2.99, 1-1.99 and 1> years to death) for both sexes in each study year. Also higher age at death (90-91 vs. 94+ years) was associated with disability. These associations remained consistent throughout the study period. This study shows that in the oldest old population both closeness to death and age at death determine the level of disability. We suggest that the complex and resource-draining care needs at the end of life will increase with growing number of people living their last years of life in very old age.

